# Development of an automated chemistry control system for secondary coolant circuits in CANDU nuclear power reactors

**DOI:** 10.1155/S1463924679000730

**Published:** 1979

**Authors:** J. R. Dean, R. B. Stewart

**Affiliations:** 1 Analytical Science Branch Atomic Energy of Canada Limited Whiteshell Nuclear Research Establishment Manitoba Pinawa R0E 1L0 Canada; 2 Powell Analytical Consulting & Service Ltd. 2023 2nd Avenue S.E. Alberta Calgary T2E 6K1 Canada


### Development of an automated chemistry control system for secondary coolant circuits in CANDU nuclear power reactors

J.R. Dean * and R.B. Stewart

*Analytical Science Branch, Atomic Energy of Canada Limited, Whiteshell Nuclear Research Establishment, Pinawa, Manitoba ROE 1LO, Canada.*

### Introduction

The CANada Deuterium Uranium-Pressurized Heavy Water nuclear power reactor (CANDU-PHW) has two separate heat transport systems, with heavy water circulating in the primary heat transport side and natural water in the secondary side. Both systems require precise chemical controls, the ultimate aim of which is to minimize metal corrosion. Corrosion on the secondary side of the steam generator could lead to boiler-tube failure and breakdown of the barrier between the two systems. While failures of boiler tubes in CANDU power reactors have been very rare, the chance of such corrosion may be greater in those reactors which will make use of saline water for condenser cooling. There is also evidence in the industry that the probability of failures increases with increasing unit size and rating. CANDU 600 boilers of the future will be larger, operate at higher temperatures and higher heat fluxes and use somewhat different construction materials than the boilers on which most of the Atomic Energy of Canada Ltd's (AECL) experience is based. Therefore, analytical chemists of the Whiteshell Nuclear Research Establishment (WNRE) undertook to devise a system which could sample, analyze and control the chemistry of a steam generator, automatically. Its development and demonstration are summarized in this article.

* *Present Address: Powell Analytical Consulting & Service Ltd., 2023 2nd Avenue, S.E., Calgary, Alberta T2E 6K1*

**Table 1. Chemical specifications for feedwater and boiler water during leaking and non-leaking condenser conditions**

Feedwater in systems using high nickel alloys for boiler tubes and copper alloys in the feedtrain or condenser:

| *Parameter* | *Permissible Range* | *Desired Value* |
|---|---|---|
| pH at 25°C | 8.8 – 9.2 | 9.1 |
| Dissolved $O_2$, µg/kg | < 5 | < 5 |
| Hydrazine, µg/kg | 10 – 20 | 10 |
| Iron, µg/kg | < 10 | < 10 |
| Copper, µg/kg | < 10 | < 10 |

Boiler water during normal operation:

| *Parameter* | *Permissible Range* | *Desired Value* |
|---|---|---|
| pH at 25°C | depends on amine solution used | |
| Free caustic, mg/kg | < 0.1 | < 0.1 |
| Dissolved $O_2$, µg/kg | < 5 | < 5 |
| Silica, mg/kg | < 5 | < 5 |
| Total dissolved solids, mg/kg | < 10 | < 10 |

Boiler water during condenser leak:

| *Parameter* | *Permissible Range* | *Desired Value* |
|---|---|---|
| pH at 25°C | 8.5 – 10.6 | 9.4 |
| Phosphate, mg/kg | 2 – 10 | 6 |
| [Na]/$CPO_4$ molar ratio | 2.2 – 2.6 | 2.3 |
| Free caustic, mg/kg | zero | zero |
| Dissolved $O_2$, µg/kg | < 5 | < 5 |
| Fluoride & chloride, mg/kg | < 75 | as low as possible |
| Total dissolved solids, mg/kg | < 40 | < 40 |

### Theory of boiler-tube corrosion and treatment

The two fundamental requirements for accelerated corrosion of boiler-tubes are (1) impurities in the boiler water which become corrosive when concentrated, and (2) an impurity-concentrating mechanism. Water chemistry conditions in certain regions of a steam generator can deviate substantially from the bulk-water chemistry specifications. For example, should boiling occur within a porous deposit on a tube, a concentrating mechanism is provided by evaporation and retardation of diffusion. In such situations, the presence

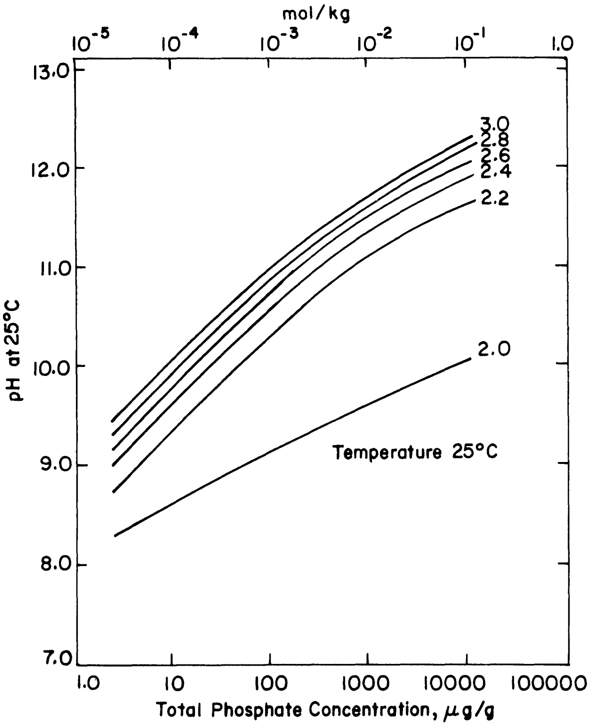

*Figure 1. Relationship of pH, orthophosphate concentration and $Z_{phos}$, at 25°C.*

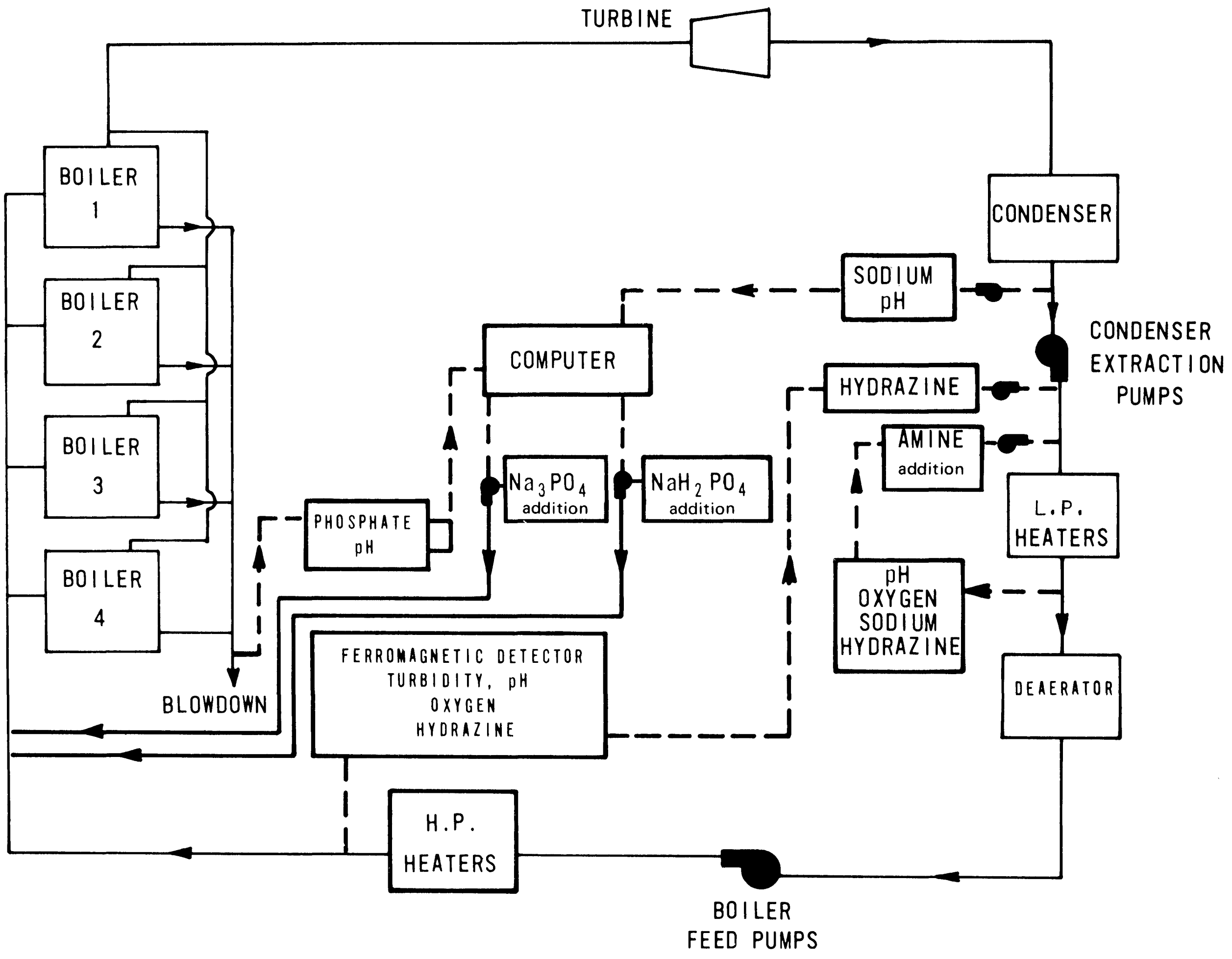

*Figure 2. Schematic of automatic chemistry control system proposed for a CANDU 600 steam generator. Solid lines indicate fluid flowlines, broken lines indicate electrical signals.*

of strong solutions of sodium hydroxide has been found to cause 'caustic embrittlement'. Acidic conditions are equally undesirable.

Minimization of boiler-tube corrosion is achieved, according to one school of thought, by maintaining high purity of feedwater and boiler water. Maintenance of high purity reduces formation of deposits on the tubes or support plates and eliminates species that become corrosive when concentrated. According to a second view, it is impractical to totally prevent potentially corrosive species from entering the boiler water, and it is thus feasible only to counteract the effects of such species by adding a non-corrosive buffering chemical such as sodium phosphate. This is beneficial in that it causes calcium or magnesium ions to precipitate as phosphates rather than to deposit on the tubes as sulphate or silicate scales. The phosphates are less adherent than sulphates and silicates, and can be more easily removed by blowdown.

### AECL approach to steam-generator chemistry control

Both of the above approaches have merit, and AECL have decided to incorporate both into the chemistry control of the CANDU 600 secondary water. In the absence of a condenser leak, the chemical control will be by the 'zero solids', or 'all volatile treatment (AVT)' mode (Table 1). During this treatment, the pH of the feedwater is adjusted by addition of a volatile amine such as cyclohexylamine. A low level of dissolved oxygen in the feedwater is ensured by the addition of hydrazine which is also volatile.

During AVT, the boiler is unprotected if ingress of potentially corrosive chemicals should occur. For example, if a seawater-cooled condenser should leak, the system would be incapable of altering the tendency of the boiler water toward acidic conditions. In this situation the alternative to In simplest terms, this is defined as the ratio of moles of sodium associated with the orthophosphate species and $OH^-$ ions, to moles of orthophosphate. Laboratory experiments carried out in the Soviet Union and the United States indicate that a $Z_{phos}$ value of between 2.2 and 2.85 is desirable. AECL specifications require the maintenance of 2 to 10 mg/kg orthophosphate and a $Z_{phos}$ value of 2.2 to 2.6. Variations outside this range can either lead to corrosive conditions or a phenomenon known as 'phosphate hideout'.

CPT is maintained by phosphate additions based upon analytical measurements of the orthophosphate content, and on determination of the $Z_{phos}$. The $Z_{phos}$ is determined from the pH of the boiler-water at 25°C and the orthophosphate concentration. A relationship exists among these three variables (Figure 1).

### Program for the development of an automated system

Automatic analysis and control using continuous on-line sensors are potentially more rapid, precise and reliable than manual control using data provided by a laboratory. The two main requirements of such an automated system are (1) an ability to control the chemistry in AVT mode, and (2) the sensitive and reliable detection of a condenser leak and, if the leak is large enough, the initiation of automatic control of the boiler using CPT. It is also desirable to provide an ability to log data and diagnose system behaviour, with the added advantage of giving the chemist a summary of past steam-generator conditions at regular intervals or upon demand.

Figure 2 shows a simplified version of the design concepts of the chemistry control system for a CANDU 600 steam generator. A central, dedicated minicomputer is the basic component. Chemical analysis information is fed to it by on-line analyzers located at strategic points on the boiler circuit. In general commercially available industrial process analysers were used. Extensive evaluation however was carried out prior to final choice of instrumentation. For rapid phosphate determination a system designed using the Technicon Analyzer principles was used. Details of the analyzer system have been presented elsewhere. Depending on the information from these analyzers, the computer actuates valves and pumps which control the addition of appropriate chemicals at the correct places. For example, operation of the phosphate system is such that the automatic addition of phosphate to the boiler will begin when a cooling-water leak is confirmed by a signal from the sodium analyzers. The computer controls the phosphate concentration, and the $Z_{phos}$ set-points, by feedback, on the basis of information supplied by phosphate and pH analyzers on the composite boiler blowdown line. In addition to initiating CPT control, the computer can log and display data, interpret alarms, and execute preventive action to correct abnormal system conditions. It was initially perceived that the feed water hydrazine and pH control would be analog-based and would be independent of the computer. In the actual event both were placed under direct digital control by the computer.

Prior to installation of a prototype in a CANDU station, it was desirable to test several aspects of the design. Saskatchewan Power Corporation expressed interest in such development work. At their Boundary Dam station (Estavan, Canada) two older boilers, Units 1 and 2, are operated continuously in CPT chemistry and three others, Units 3, 4 and 5, are operated continuously in AVT chemistry. A co-operative program was undertaken to establish the confidence necessary to proceed with a prototype nuclear station installation.

For reasons of effort and economy, it was believed unnecessary to demonstrate a complete chemistry control system at Boundary Dam. Key elements were therefore selected for testing. These included the computer, control algorithm, sodium analyzers for leak detection, and on-line phosphate and pH instruments. In addition, commercial dissolved oxygen analyzers were compared, hydrazine mixing and reaction rates were studied and a hydrazine analyzer was tested on-line with feedback control. The on-line calibration of analyzers, and data display and print-out were also selected for demonstration. Concurrently a rapid on-line procedure for phosphate analysis was developed.

A block diagram of the Boundary Dam Station test arrangement is shown in Figure 3. Since Boundary Dam is a lignite coal-fired station with dust and fly ash prevalent, the location and protection of the analyzers and computer were carefully considered. The computer was housed in a clean, air-conditioned room serving other station instrumentation. It was connected by communication cables to the analyzer rooms of Units 4 and 5, and to a temporary shelter, housing the phosphate and pH analyzer at Unit 1. The hydrazine

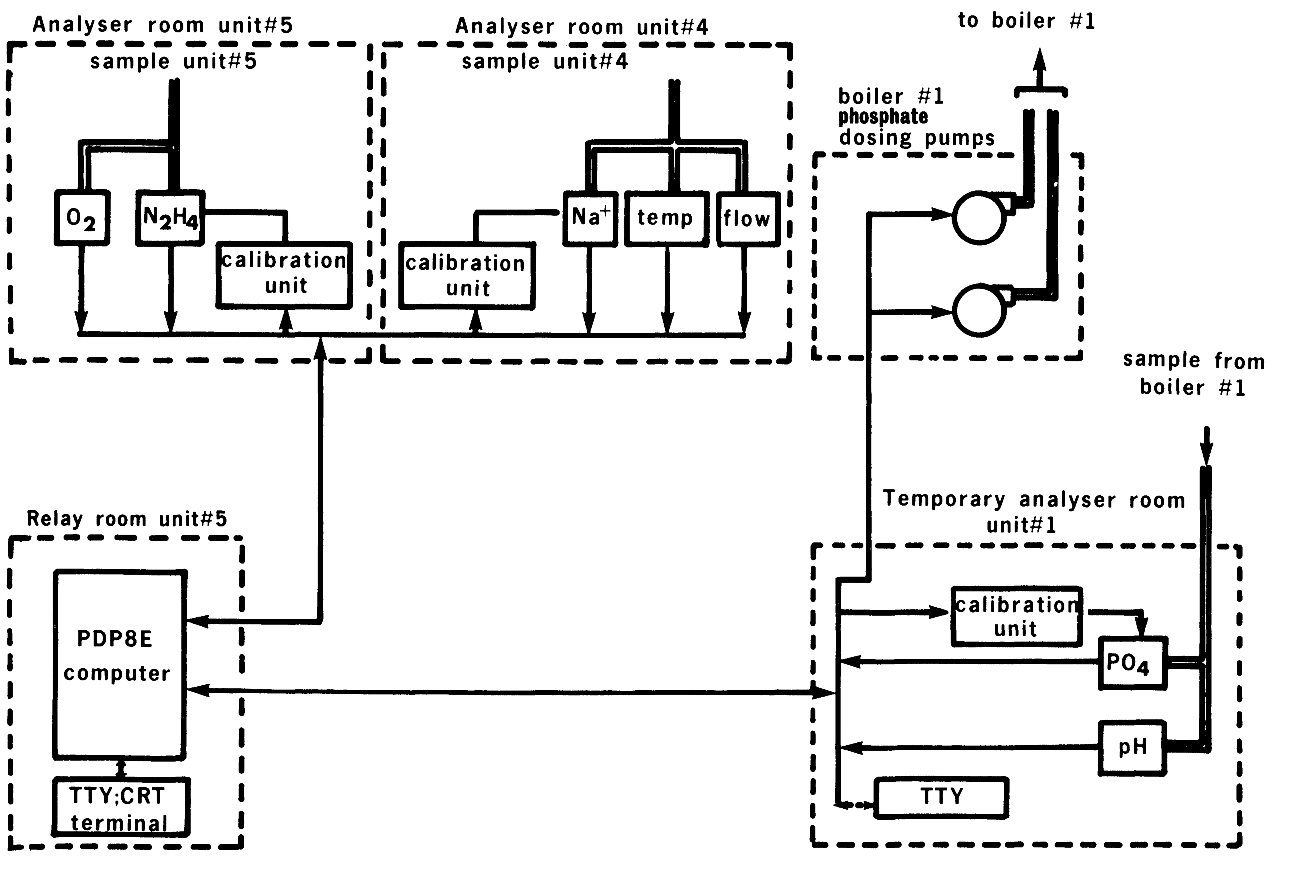

*Figure 3. Schematic of the Boundary Dam Station demonstration lay-out. TTY= teletype.*

and dissolved oxygen analyzers were located in another clean instrument room. Data transmission lines of up to several hundred feet in length were used successfully.

The computer had three tasks: data-logging, CPT selection, initiation and control, and automatic analyzer calibration. The data-logging mode included sampling data from all analyzers once per minute and checking for alarm conditions. The data collected at one minute intervals were examined and discarded, except that at five-minute intervals they were displayed on the screen, typed out on reports and stored in the computer files. In Figure 4, the computer values of sodium ion (μg/kg) dissolved oxygen (μg/kg), hydrazine (μg/g), phosphate (μg/g) and pH are shown for three time periods of 11 May, 1977. Also included in the listing is the $Z_{phos}$ value for the Unit 1 boiler and the temperature of the sample delivered to the sodium ion monitor at these times. The number of significant figures is not an indication of precision and would be 'rounded-off' to three in a prototype installation. Similarly, the exponential notation would be converted to a floating point format. The stored data were typed three times daily, to provide a summary report.

```
MONTR       18:29       05/11/77
------------------------------------------
NA+   1.1269E00
OXYGN      7.8274E00
HYDRZ           1.1724E01
ZPHOS                2.3166E00
PO4                       9.8372E00
PH                             9.4985E00
TEMP                                6.0873E01

MONTR       18:33       05/11/77
------------------------------------------
NA+   1.1196E00
OXYGN      7.7689E00
HYDRX           1.1551E01
ZPHOS                2.3103E00
PO4                       9.8772E00
PH                             9.4918E01
TEMP                                6.0873E01

MONTR       18:38       05/11/77
------------------------------------------
NA+   1.1124E00
OXYGN      7.7104E00
HYDRZ           1.1551E01
ZPHOS                2.3245E00
PO4                       9.9173E00
PH                             9.5119E00
TEMP                                6.0873E01
```

*Figure 4. Typical five-minute report showing the concentrations (μg/kg) of sodium ion in Unit 4, dissolved oxygen and hydrazine in Unit 5 feedwater and levels of ZPHOS, orthophosphate (μg/g) and pH in Unit 1 boiler. The temperature (°F) refers to that of the sample flowing to the sodium ion analyzer.*
*'ZPHOS' refers to [Na]$CPO_4$, the ratio of orthophosphate-derived sodium ion concentration to the sum of phosphate ion concentrations.*

```
MONTR       15:50       05/12/77
------------------------------------------
NA+   1.1708E00
OXYGN      6.3064E00
HYDRZ           4.4090E01
ZPHOS                2.4119E00
PO4                       1.1584E01
PH                             9.6799E00
TEMP                                5.9287E01

MONTR       15:55       05/12/77
------------------------------------------
NA+   1.1632E00
OXYGN      6.3064E00
HYDRZ           4.4090E01
ZPHOS                2.4167E00
PO4                       1.1626E01
PH                             9.6867E00
TEMP                                5.9409E01

MONTR       16:00       05/12/77
------------------------------------------
```

| TIME | NA+ | OXYGN | HYDRZ |
|---|---|---|---|
| 06:00 | 1.2499E00 | 7.1644E00 | 1.6234E01 |
| 05:05 | 1.2499E00 | 7.0279E00 | 1.6284E01 |
| 05:10 | 1.2654E00 | 7.2229E00 | 1.6284E01 |
| 05:15 | 1.2810E00 | 6.9889E00 | 1.6055E01 |
| 06:20 | 1.2654E00 | 7.0474E00 | 1.6455E01 |
| 05:25 | 1.2423E00 | 7.0864E00 | 1.6055E01 |
| 05:30 | 1.2423E00 | 7.0669E00 | 1.6055E01 |
| 06:35 | 1.2576E00 | 7.0474E00 | 1.6055E01 |
| 06:40 | 1.2654E00 | 7.0669E00 | 1.6055E01 |
| 05:45 | 1.2576E00 | 7.0279E00 | 1.6055E01 |
| 06:50 | 1.2423E00 | 6.9499E00 | 1.6055E01 |
| 06:55 | 1.2122E00 | 6.9694E00 | 1.6055E01 |
| 07:00 | 1.2272E00 | 6.9499E00 | 1.5825E01 |
| 07:05 | 1.2576E00 | 6.9304E00 | 1.5825E01 |
| 07:10 | 1.3128E00 | 6.9499E00 | 1.5825E01 |
| 07:15 | 1.3372E00 | 6.9374E00 | 1.5825E01 |
| 07:20 | 1.3128E00 | 7.0084E00 | 1.5591E01 |
| 07:25 | 1.2654E00 | 6.9109E00 | 1.5825E01 |
| 07:30 | 1.2576E00 | 6.9694E00 | 1.5825E01 |
| 07:35 | 1.2810E00 | 6.9889E00 | 1.5596E01 |
| 07:40 | 1.2810E00 | 6.9304E00 | 1.5596E01 |
| 07:45 | 1.3048E00 | 7.0084E00 | 1.5366E01 |
| 07:50 | 1.2889E00 | 6.9499E00 | 1.5596E01 |
| 07:55 | 1.2810E00 | 7.2229E00 | 1.5366E01 |
| 08:00 | 1.3048E00 | 6.9499E00 | 1.5366E01 |

*Figure 6. Portion of typical eight-hour report showing Congruent Phosphate control behaviour (Set Z refers to $Z_{phos}$ setpoint and Set P refers to the orthophosphate setpoint).*
*'ZPHOS' = [Na]/$CPO_4$.*

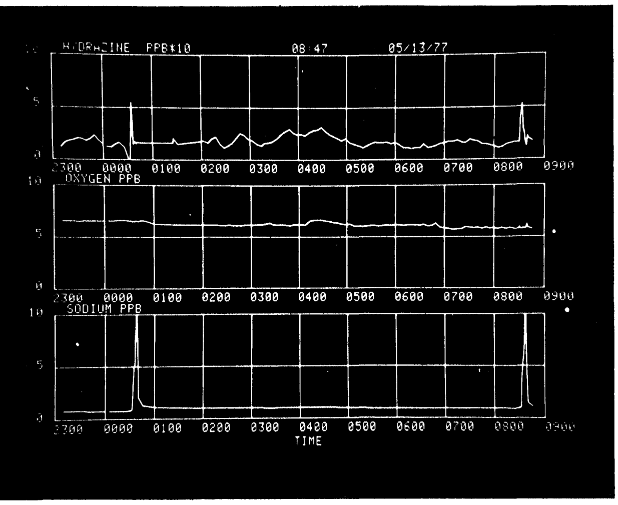

*Figure 5. A typical CRT display showing the behaviour of hydrazine, dissolved oxygen and sodium ion over a ten-hour period: 'ppb' indicates μg/kg.*

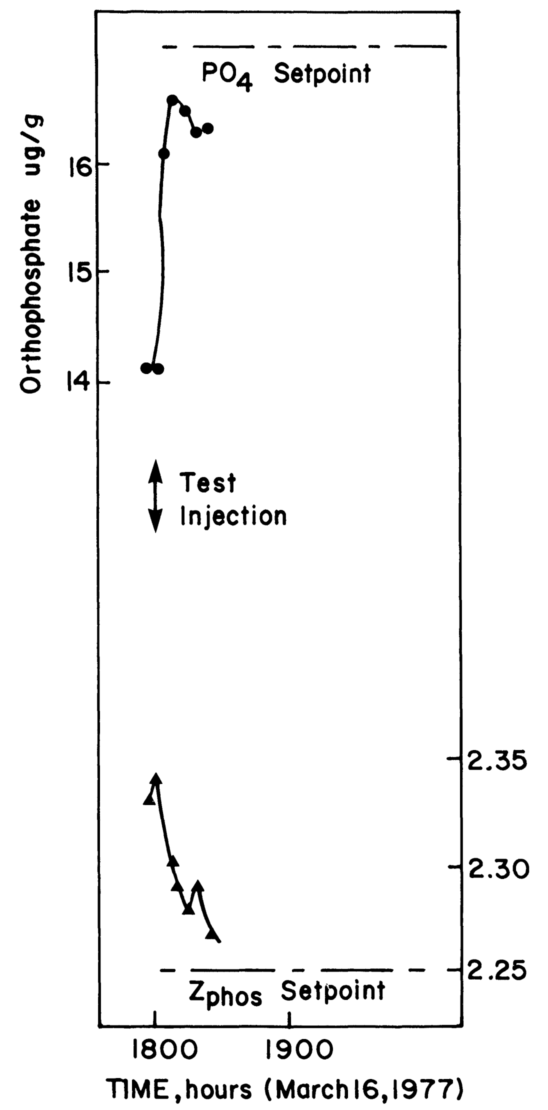

*Figure 7. Illustration of the behaviour of the Congruent Phosphate Treatment control system during an injection test. (Increasing $Z_{phos}$, no blowdown).*
*$Z_{phos}$ = $[Na]/CPO_4$, the ratio of orthophosphate-derived sodium ion concentration to the sum of phosphate ion concentrations.*

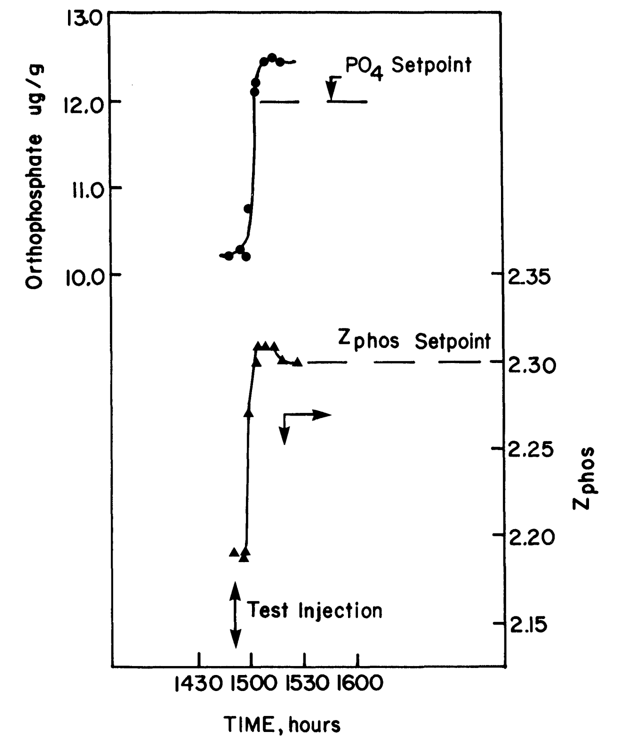

*Figure 8. Illustration of the behaviour of the Congruent Phosphate Treatment control system during an injection test. (Decreasing $Z_{phos}$, no blowdown).*

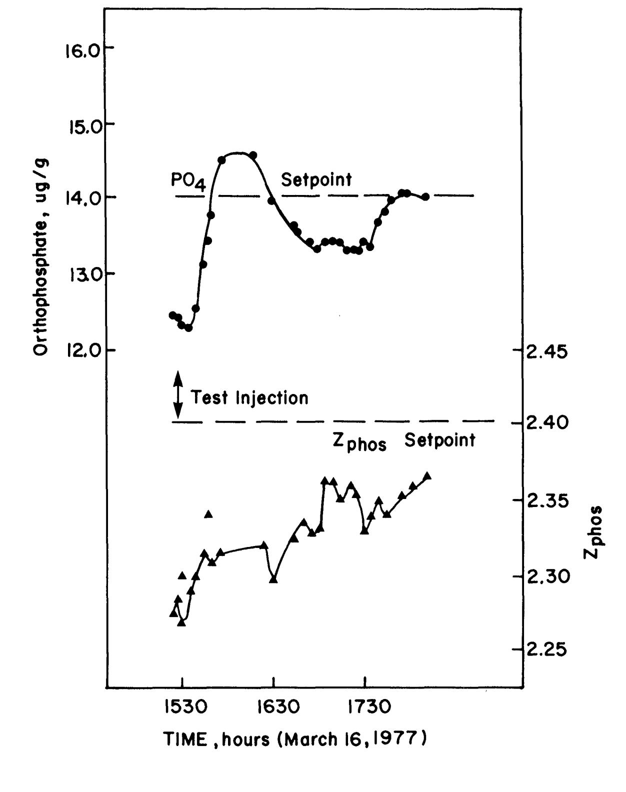

*Figure 9. Illustration of the behaviour of the Congruent Phosphate Treatment control system during an injection and controlled climb test.*

In the CANDU prototype, sodium analyzers would monitor the condensate water and the computer would initiate congruent phosphate treatment upon confirmation of a leak (4.5 L/h of sea water was taken as the maximum tolerable leak rate). For the Boundary Dam tests, it was necessary to simulate a leak by means of an electrical signal. (This meant that the sodium analyzer tests need not necessarily be done on the boiler under phosphate control, and it was convenient to install these analyzers on Unit 4 which operates in the AVT mode.) When this was done, the computer demonstrated its ability to confirm the indication using its leak detection logic, then bring the boiler into congruent phosphate chemical control by initiating the operation of monosodium phosphate and disodium phosphate dosing pumps. Data from three of the analyzers (sodium ion, dissolved oxygen and hydrazine) could be displayed on a cathode ray tube, as illustrated in Figure 5.

The deviations observable at 0030 and 0830 hours are artefacts of the automatic calibration process. In a reactor installation it is foreseen that all analyzer data could be displayed in this way upon demand. Figure 6 shows the format of the automatic CPT control summary report. The last two columns indicate the $Z_{phos}$ set-point (SET Z) and the phosphate set-point (SET P) during the time interval reported.

Figures 7-11 show the results of several tests that were conducted. In each figure, the upper plot is of the boiler water orthophosphate concentration with its scale on the left, and the lower plot is of the boiler water $Z_{phos}$ with its scale on the right. The time-of-day scale is across the bottom. The set-point values are marked with horizontal dashed lines and the start of each test is indicated by a vertical arrow. It should be recalled that since boilers # 1 and #2 at Boundary Dam station operate continuously under CPT conditions, our tests did not start at a zero $PO_4$ concentration but at some finite small value.

In the first test, shown in Figure 7, an initial rapid injection into the boiler was performed. The conditions in the boiler before the test began were a phosphate concentration of 10.2 $\mu$g/g and a $Z_{phos}$ of 2.19. The setpoints to be reached were 12.0 and 2.30 $\mu$g/g respectively. Within about 15 min, the setpoint levels were reached after an overshoot of not more than 0.5 $\mu$g/g in phosphate level and 0.01 in the $Z_{phos}$. For the CANDU prototype, the permissable error between the actual boiler conditions and the setpoint levels is ±2 $\mu$/g for phosphate and ±0.1 for $Z_{phos}$.

The next test was also an initial injection (Figure 8). In this test a decrease in the $Z_{phos}$ level from its initial value of 2.33 to a set-point at 2.25 was required. The phosphate level was to be increased to 17 $\mu$g/g from 14 $\mu$g/g. Again the injection was very rapid and the change was established to within 0.5 $\mu$g/g and 0.02 $Z_{phos}$ after about 20 minutes.

Figure 9 illustrates a situation in which the initial injection failed to change the $Z_{phos}$ value to the setpoint level and the controller took over. The starting conditions were 12.2 $\mu$g/g phosphate and $Z_{phos}$ 2.27. During the course of this test, it was known that several disturbances were acting upon the boiler. As intended by its design, the controller detected these and managed within 2.5 hours to bring the phosphate level to its set point and come within 0.02 of the $Z_{phos}$ setpoint.

Figure 10 shows the results of a test in which there was no blowdown. Identifiable disturbances were imposed by causing

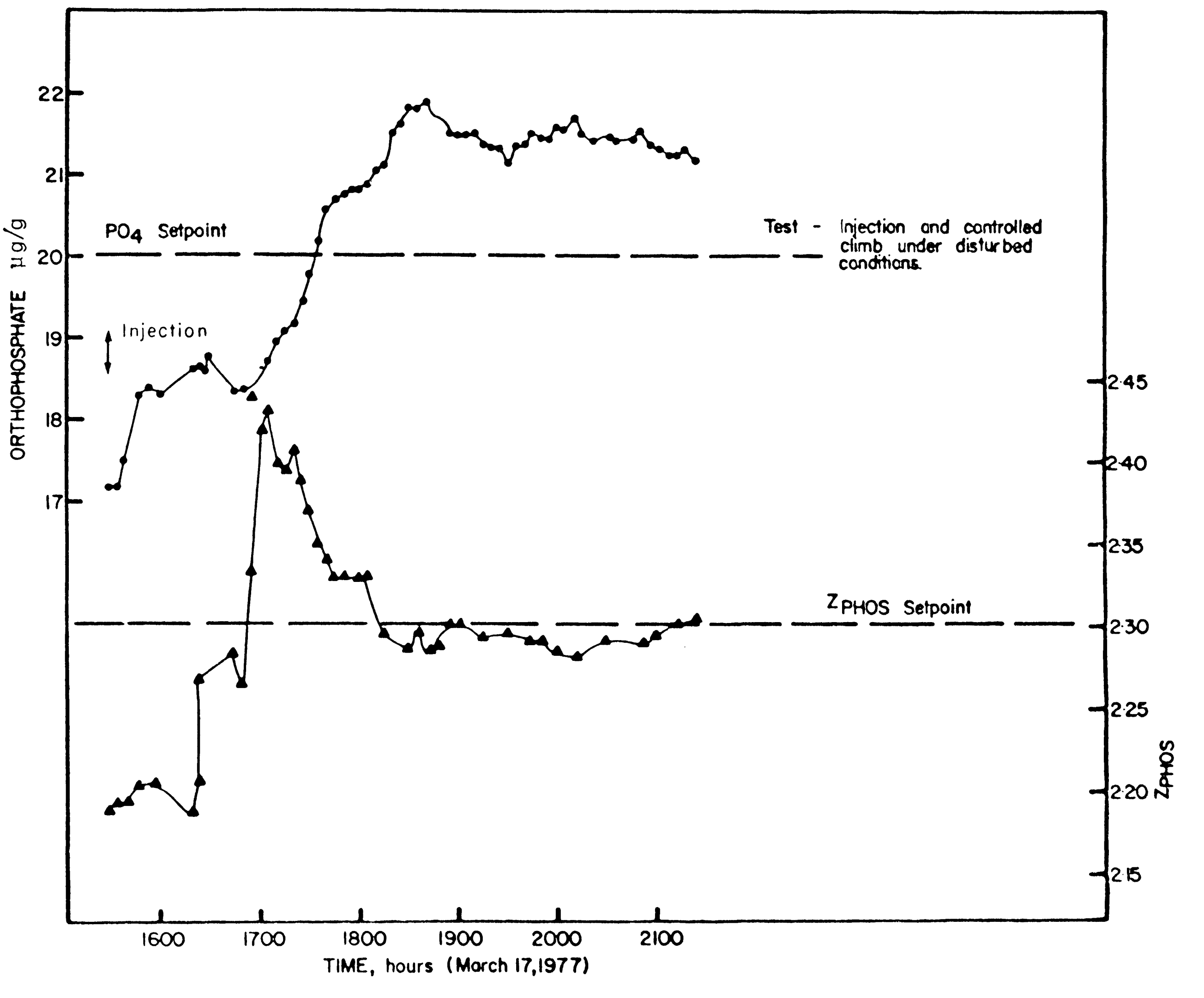

*Figure 10. Illustration of the behaviour of the Congruent Phosphate Treatment control system during injection and controlled climb testing. (Controlled disturbances were introduced and boiler blowdown was inoperative during this test).*

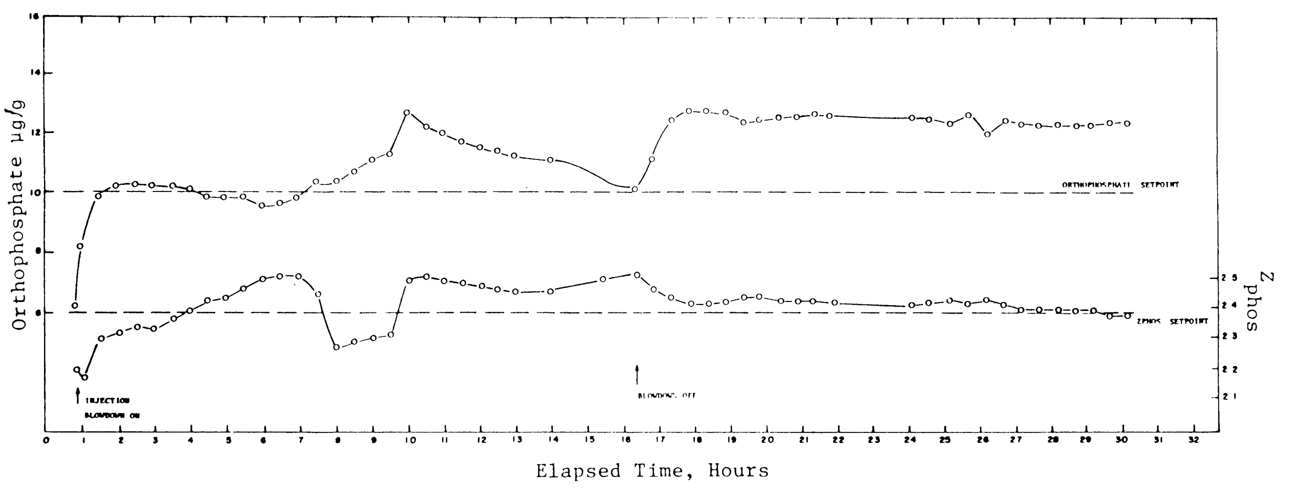

*Figure 11. Illustration of the long-term ability of the CPT control system to maintain $PO_4$ and $Z_{phos}$ setpoints.*

the controller to believe that the phosphate pump rates were larger than they in fact were. The initial conditions were 17 µg/g phosphate and 2.2 $Z_{phos}$. The setpoints were 20 µg/g phosphate and 2.30 $Z_{phos}$. The initial injection failed to cause the setpoints to be reached. However, the controller eventually adjusted to the 'disturbances' and brought the $Z_{phos}$ to within 0.01 of its setpoint. The controller allowed the phosphate level to overshoot by 1µg/g because of the lack of blowdown and as a consequence of adjusting the $Z_{phos}$.

Figure 11 illustrates the long-term ability of the CPT control system to maintain the $PO_4$ and $Z_{phos}$ setpoints. Under controlled climb conditions the boiler chemistry was to go from initial conditions of 8µg/g phosphate and $Z_{phos}$ 2.3 to setpoints of 10µg/g phosphate and 2.4 $Z_{phos}$. While the boiler blowdown was in continuous operation, the setpoints were reached after two hours. After 16 hours, the blowdown was inadvertently cut off. Up to that time, it was counteracting the tendency of the boiler to increase in phosphate level while trying to adjust the $Z_{phos}$ precisely. After the shut-off of the blowdown, the tendency of the controller to adjust the $Z_{phos}$ precisely resulted in a 1.5 µg/g overshoot on the phosphate level setpoint. However, throughout the test, the $Z_{phos}$ value was maintained for 24 hours within 0.03 of the setpoint and the phosphate level was maintained close to the ± 2µg/g specification.

All analyzers in the system for a CANDU prototype installation would have temperature and flow monitor and alarms, and automatic calibration. In the Boundary Dam tests, the alarms were demonstrated for the sodium analyzer only, while three analyzers (hydrazine, sodium and phosphate) were automatically calibrated. In response to computer signals, the calibration unit sequentially fed the appropriate analyzer a sample to each of two standard solutions. The analyzer response to these standards was used by the computer to calculate a calibration scale for conversion of subsequent sample signals to the correct corresponding chemical concentrations and units.

### Summary of results of the development work

Upon completion of the experimental program at Boundary Dam, the chemistry control was successfully demonstrated in operation, to the representatives of several Canadian electrical utilities. It was clear that such a system is of interest to operators of both nuclear- and fossil-fuelled power stations and that a technical foundation has been established which permits progression to a prototype installation.

The results of the program can be summarised in the following sentences:

Hydrazine concentrations could be maintained automatically and dissolved oxygen levels controlled. The computer algorithm was appropriate for control in either AVT or CPT modes and for the selection of CPT upon detection of a condenser leak. The system could control the boiler's chemistry within set limits for an extended period of time. Automatic calibration of the on-line analyzers could be done successfully. The data-logging, printout and display functions of the computer were successful and an aid to good chemistry management.

ACKNOWLEDGEMENT

This project was the work of many people of Saskatchewan Power Corporation and Atomic Energy of Canada Limited. We wish particularly to acknowledge the contributions of Messrs. B. Baker, A. Whyte, R. Portman, M. Myers, J. Westdal and G. Young.

